# Exhaustion of CD4+ T-cells mediated by the Kynurenine Pathway in Melanoma

**DOI:** 10.1038/s41598-019-48635-x

**Published:** 2019-08-21

**Authors:** Soudabeh Rad Pour, Hiromasa Morikawa, Narsis A. Kiani, Muyi Yang, Alireza Azimi, Gowhar Shafi, Mingmei Shang, Roland Baumgartner, Daniel F. J. Ketelhuth, Muhammad Anas Kamleh, Craig E. Wheelock, Andreas Lundqvist, Johan Hansson, Jesper Tegnér

**Affiliations:** 1 Unit of Computational Medicine, Department of Medicine, Centre for Molecular Medicine, Karolinska Institute, SE-171 76 Stockholm, Sweden; 2 Algorithmic Dynamics Lab, Unit of Computational Medicine, Department of Medicine Solna, Centre for Molecular Medicine, Karolinska Institute and SciLifeLab, SE-171 77 Stockholm, Sweden; 3 Department of Oncology-Pathology, Karolinska University Hospital, Stockholm, Sweden; 4 Experimental Cardiovascular Research Group, Cardiovascular Medicine Unit, Centre for Molecular Medicine, Department of Medicine, Karolinska Institute, Karolinska University Hospital, SE-171 76 Stockholm, Sweden; 5 Division of Physiological Chemistry II, Department of Medical Biochemistry and Biophysics, Karolinska Institute, SE-171 77 Stockholm, Sweden; 6 Department of Genomics and Bioinformatics, Positive Bioscience, Mumbai, -400 002 India; 7 Biological and Environmental Sciences and Engineering Division (BESE), Computer, Electrical, and Mathematical Sciences and Engineering Division (CEMSE), King Abdullah University of Science and Technology (KAUST), Thuwal, 23955–6900 Saudi Arabia; 8 Department of Immunology, Genetics & Pathology, Science for Life Laboratory, Uppsala University, Uppsala, Sweden

**Keywords:** Tumour immunology, Immunosurveillance, Network topology

## Abstract

Kynurenine pathway (KP) activation by the enzymatic activity of indoleamine 2,3-dioxygenase1 (IDO1) and kynurenine (KYN) production represents an attractive target for reducing tumour progression and improving anti-tumour immunity in multiple cancers. However, immunomodulatory properties of other KP metabolites such as 3-hydroxy kynurenine (3-HK) and kynurenic acid (KYNA) are poorly understood. The association of the kynurenine metabolic pathway with T-cell status in the tumour microenvironment were characterized, using gene expression data of 368 cutaneous skin melanoma (SKCM) patients from the TCGA cohort. Based on the identified correlations, we characterized the production of KYN, 3-HK, and KYNA *in vitro* using melanoma-derived cell lines and primary CD4+ CD25− T-cells. Activation of the CD4+ T-cells produced IFNγ, which yielded increased levels of KYN and KYNA. Concurrently, kynurenine 3-monooxygenase (KMO) expression and proliferation of CD4+ T-cells were reduced, whereas exhaustion markers such as PD-L1, AHR, FOXP3, and CTLA4 were increased. Additionally, an analysis of the correlation network reconstructed using TCGA-SKCM emphasized KMO and KYNU with high variability among BRAF wild-type compared with V600E, which underscored their role in distinct CD4+ T-cell behavior in tumour immunity. Our results suggest that, in addition to IDO1, there is an alternative immune regulatory mechanism associated with the lower KMO expression and the higher KYNA production, which contributes to dysfunctional effector CD4+ T-cell response.

## Introduction

Melanoma is the most fatal forms of skin cancer arises from the malignant transformation of the melanocytes of which cutaneous melanoma is the most common form^1,2^. The five-year relative survival rate for patients with stage I melanoma is more than 95%, but it dramatically decreases to 10% for those with stage IV disease^3^. Besides, melanoma is considered to be an immunogenic tumour due to the elevated level of somatic mutations and tumour antigen-specific T-cells, and these features have made immunotherapy a promising treatment for these patients^4^. Although the utilization of immune checkpoint inhibitors (ICIs) such as antibodies against CTLA4 and PD-1/PD-L1 has revolutionized the treatment of melanoma in advanced stages, only a subset of patients shows long-lasting responses to this treatment. It is reported that ICIs block different immunomodulatory pathways, leading to an increase in CD4+, CD8+ T-cells and a reduction in regulatory T-cells^5,6^. In recent years, complementary immuno-metabolism which targets Indoleamine 2 3-Dioxygenase1 (IDO1), has been used to improve response rates of ICIs. However, IDO1 blockade and PD-1 inhibitor combination treatment in metastatic melanoma have not shown any improvement in survival compared with single PD-1 inhibitors treatment^7^. CD8+ and CD4+ T-cells, commonly known as cytotoxic T-cells and helper T-cells (TH), are fractions of white blood cells, which are typically attributed to the cellular immune responses that protect against tumours^8^. The complex role of CD4+ T-cells in anti-tumour immunity reveals various functions of several types of CD4+ T-cells^9^. TH1 cells are characterized by the secretion of cytokines such as IFNγ and TNFα. The superior anti-tumour effect of TH1 cells is reflected by a large amount of IFNγ secretion, which is responsible for priming and enhancing CD8 T-cell response^9^. On the other hand, specific subsets of CD4 T-cells such as regulatory T-cells (T_regs_), which are characterized by the expression of the FOXP3 transcription factor, have tumour-promoting activities. It has been shown that the level of T_regs_ is augmented in a different type of cancer such as melanoma, where they prevent anti-tumour immune responses^10^.

The tryptophan (TRP) degradation along the kynurenine pathway (KP) plays a critical role in the regulation of immune response^11^. The significance of KP activation depends on the chains of enzymatic reaction and production of active metabolites such as kynurenine (KYN), kynurenic acid (KYNA), qunilic acid (QUIN) or anthranilic acid (AA). Three major phases of KYN metabolic pathway which lead to 3-HK, AA and KYNA production are mediated by the enzymatic activity of kynurenine 3-monooxygenase (KMO), kynureninase (KYNU) and kynurenine aminotransferase (CCBL1, AADAT, CCBL2, and GOT2, collectively known as KATs), respectively. Among KP metabolites, KYN production has been linked to cancer immune escape and is primarily explained by the role played by IDO1 inhibition which has emerged as a potential candidate in the context of cancer immune therapy^12–17^. The deregulation of the KP causes an alteration of the balance between KYNA and QUIN, as defined in many neurological and inflammatory disorders^18,19^. Although KYNA alteration is considered to be a potential marker in cancer patients, the role played by KYNA dysregulation is not clearly understood in this context.

Moreover, the immunosuppressive mechanism of IDO1 can be explained primarily by three effector pathways, including GCN2 activation and mTOR inhibition, which leads to TRP deprivation and AHR pathway activation with KYN^20–24^. TRP depletion leads to reduced proliferation and increased apoptosis of T-cells. More recently, this concept was developed by the identification of additional KP metabolites, which resulted in the de novo production of T_regs_ from CD4+CD25− naïve T-cells^25^. More importantly, AHR, which is kynurenine agonist, is recognized as a crucial factor in the regulation of TH cell subsets, and it controls the IDO1 expression^26,27^. Furthermore, it is shown that AHR deficiency leads to smaller T_regs_ population and elevated levels of Th1 and Th17^28^. KYN itself provides the substrate for kynurenine 3-monooxygenase (KMO) and kynurenine aminotransferase (KAT) which convert KYN into 3-hydroxykynurenine (3-HK) and KYNA (kynurenic acid)^29,30^. KYNA is also regarded as an endogenous ligand of AHR and functional implication of elevated KYNA is tightly controlled by AHR activation. On the other hand, KMO controls the Th17 differentiation by limiting the continuous exposure to AHR^31,32^. Therefore, it is of interest to investigate if the AHR/KYNA/KMO axis is involved in the regulation of the TH cell subset.

In this study, we characterized the association of the kynurenine metabolic pathway with T-cell status in the tumour microenvironment, using gene expression data of cutaneous skin melanoma (SKCM) patients from the TCGA cohort. In this respect, based on the identified correlations, we characterized the production of KYN, 3-HK, and KYNA *in vitro* using melanoma-derived BRAF wild type (wt) and BRAF V600E mutant cell lines cultured with primary CD4+ CD25− T-cells. Additionally, the correlation network was analysed in order to investigate the correlation networks for CD4+ T-cells and KP-related genes in BRAF V600E compared with BRAF wt SKCM-TCGA data.

## Results

### Kynurenine pathway related genes are associated with T-cell status in the tumour microenvironment

**Figure 1:**
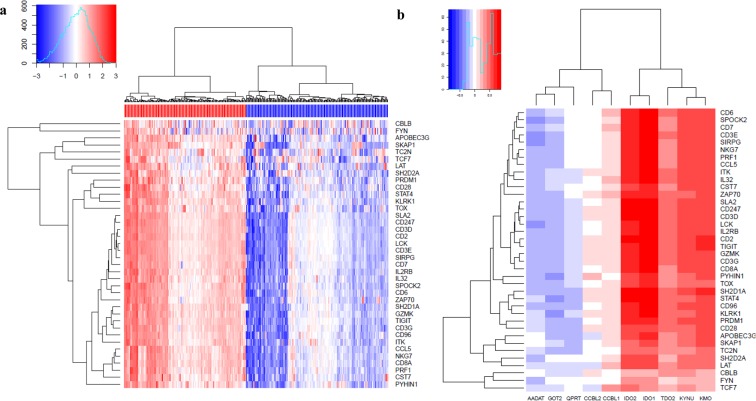
KP pathway correlates with T-cell exhaustion. **(a)** A heat map of T-cell signature genes expression from 368 metastatic melanoma patients. (**b)** A heat map of correlation between T-cell signature genes expression and KP target genes expression. (red indicates T-cell signature high, blue indicates T-cell signature low).

Tumour-infiltrating lymphocytes are considered a favorable prognostic marker in several malignancies and increased levels of TILs have been associated with clinical outcome and more prolonged survival for patients with melanoma. Therefore, to explore whether kynurenine metabolic pathway is associated with T-cell status in the tumour microenvironment, gene expression data of mRNA of 368 cutaneous melanoma metastases (SKCM) in the TCGA cohort were divided into groups with low and high expression of T-cell signature genes which has reported previously^33^ (Fig. 1a). Spearman correlation coefficient analyses were performed on kynurenine pathway-related genes (IDO1/2, TDO2, KMO, KYNU, CCBL1/2, GOT2, AADAT, and ACMSD) and T-cell status-related genes which showed that expression of IDO1, IDO2, KYNU, and KMO are associated with T-cell status-related genes (Fig. 1b, Table S3, Supplementary Fig. S4).

### Inhibition of CD4+CD25− T-cell proliferation by melanoma cell lines (MCLs) associated with KP enzymatic alteration

**Figure 2:**
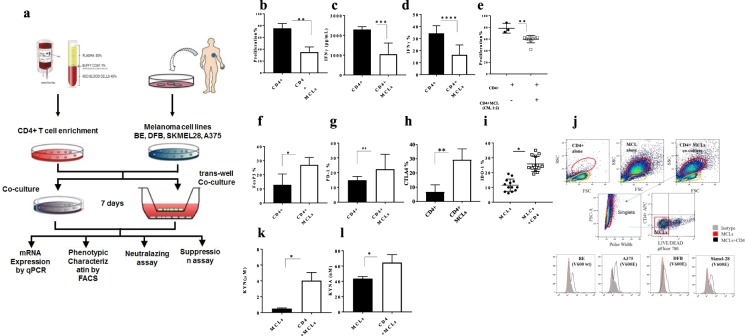
Inhibition of CD4+CD25− T-cell proliferation by MCLs associated with KP enzymatic alteration. (**a**) Schematic workflow of the experimental design (**b**) Measurement of CD4+ T-cell proliferation *in vitro* by CFSE dilution alone and in culture with MCLs (**c**,**d**) IFNγ secretion and IFNγ expression levels of CD4+ T cells in culture with MCLs by ELISA and flow cytometry (**e**) Measurement of CD4+ T-cell proliferation *in vitro* by CFSE dilution with medium (RPM1640) and with conditioned medium derived from CD4+ and MCLs co-culture (**f**–**h**) FOXP3, PD1 and CTLA4 protein expression from educated CD4+ T-cells by flow cytometry (**i**), IDO1 protein expression from educated MCLs determined by flow cytometry (**j**) Gating strategies of IDO1 positive MCLs cultured with CD4+ T-cells (**k**,**l**) KYN and KYNA production by LC-MS/MS. Graphs show individual data, and horizontal lines show mean ± s.e.m. *P ≤ 0.05, **P ≤ 0.005 by independent samples t-test, n > three biological replicates cultured with four different melanoma cell lines.

In order to characterize how melanoma tumours influence the CD4+ CD25− T-cells, healthy donors’ pre-activated primary CD4+CD25− T-cells were co-cultured with human cutaneous melanoma cell lines, including DFB, A375, and SK-MEL-28 (V600E) and BE and SK-MEL-2 (V600 wt), for five days (Fig. 2a). As expected, the proliferation and IFNγ production of CD4+ T-cells was significantly reduced when co-cultured with MCLs (Fig. 2b,d) or with supernatant harvested from MCLs (Fig. 2e). Furthermore, CD4+ T-cells had a higher expression of CTLA4 and FOXP3 in the presence of MCLs (Fig. 2f,g). Collectively, these observations may suggest the development of an exhausted CD4+ T-cell phenotype. To determine whether changes in KP metabolite may involve CD4+ T-cells exhaustion, KP metabolites concentration was measured by HILIC–MS/MS in supernatant derived from each cell type alone or co-cultured after 48 hours. This analysis showed a profound depletion of TRP, 3-HK production, and higher production KYN, KYNA in co-cultures compared with the medium from MCLs and CD4+ T-cells alone (Fig. 2k,l,k, Supplementary Fig. 1b–g).

Therefore, we then investigated whether KYN and KYNA production promotes an exhausted CD4+ T-cell phenotype upon exposure to MCLs. To this end, mRNA and protein expression of *IDO1* and mRNA expression of *IDO2*, *TDO2* which mediate the production of KYN were compared in both CD4+ T-cells and MCLs before and after co-culture (48 hours). Only higher expression of IDO1 (Fig. 2i) and higher kynurenine production were detected in co-cultures compared with MCLs alone (Fig. 2k), suggesting a higher activity of IDO1 in co-culture set-up. However, IDO2, TDO2 were not detected in these cell lines before and after exposure to CD4+ T-cells.

### IDO1 blockade repairs CD4^+^ T-cells proliferation in culture with MCLs

**Figure 3:**
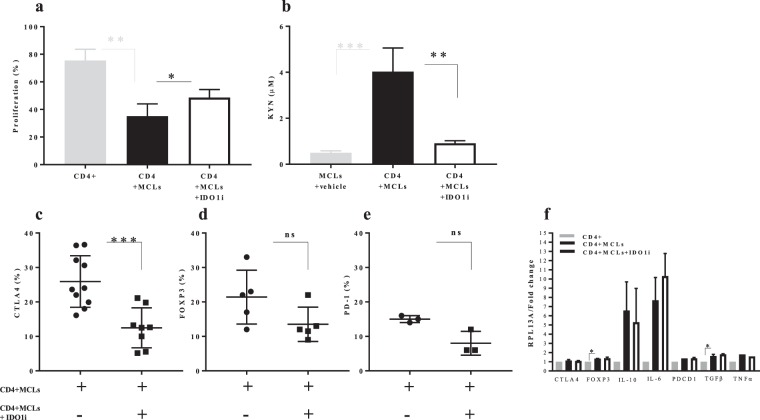
Functional consequences of IDO1 blockade on CD4+ T cell- MCLs co-culture. (**a**) The proliferation of CD4+ T-cells in the presence of MLCs alone and with the IDO1 blockade (**b**) KYN production by HPLC after 48 h and (**c**) Ratio of CTLA4+, FOXP3+ or PD-1+ population among CD4+ T-cells by flow cytometry (**d**), CTLA4, FOXP3, PD-1 IL-10, IL-6, TGFβ and TNFα mRNA expression after five days were analysed by real-time PCR and normalized to RPL13A in CD4+ T-cells. IDO1i: IDO1 inhibitor (INCB024360). Graphs show individual data, and horizontal lines show mean ± s.e.m. *P ≤ 0.05, **P ≤ 0.005, ***P ≤ 0.0005 by One-Way ANOVA (non-parametric) and independent samples t-test (two-sided) and the Tukey multiple comparison procedure was used to determine significance; and adjusted P values for the differences in group means can be seen in Table S6, n > three biological replicates cultured with four different melanoma cell lines.

To further investigate whether IDO1 blockade and KYN depletion can recover CD4+ T-cell proliferation, MCLs (BE, DFB, A375, and SK-MEL-28) were cultured for 48 hours with CD4+ T-cells in the presence or absence of IDO1 inhibitor INCB024360 (Epacadostat). CD4+ T-cells proliferation was recovered in culture with MCLs (Fig. 3a) while the IDO1 inhibition led to lower KYN production in co-culture set-up. IDO1 inhibition also led to lower CTLA4 protein expression from educated CD4+ T-cells (Fig. 3b,c), although the mRNA levels of cytokines did not differ significantly upon IDO1 blockade (Fig. 3d–f). Interestingly, CD4+ T-cells were more proliferative in a BRAF wt cell line compared with V600E cell lines in the presence of the IDO1 blockade (Supplementary Fig. 3b) which may suggest the possible relation between mutation status and KP metabolites in district CD4+ T-cell behavior in co-culture set-up.

### CD4+ T-cell behavior in co-culture set-up is linked to KYNA production

**Figure 4:**
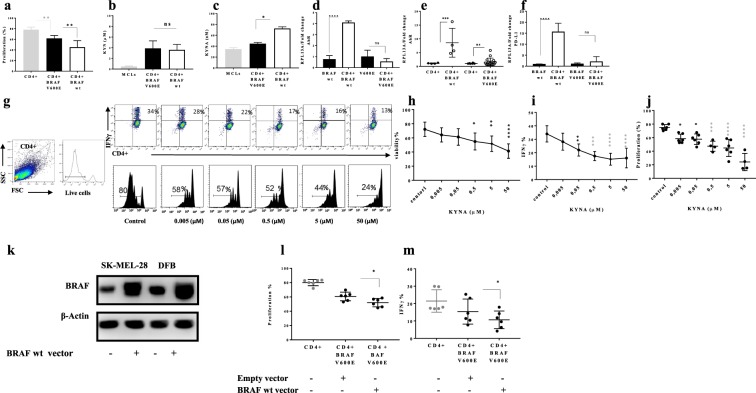
The district CD4+ T-cell behaviour in co-culture set-up links to KYNA production. (**a**) The proliferation of CD4+ T-cells in the presence of V600E and V600 wt MLCs (**b**,**c**) KYN and KYNA production by HPLC after 48 h in co-culture set-up (**d**) AhR mRNA expression in V600E and V600 wt MLCs (**e**) CD4+ T-cells in co-culture set-up (**f**) PD-L1 mRNA expression in V600E and V600 wt MLCs in after 5 days co-culture set-up were analysed by real-time PCR and normalized to RPL13A in CD4+ T-cells (**g**) show representative images of gating strategy (left panel), IFNγ production (right upper panel) and CD4+ T-cell proliferation (right lower panel) in different concentrations of KYNA (**h)** Cell toxic concentration of KYNA was defined using viability assay by flow cytometry (**i,j)** CD4+ T-cell proliferation and IFNγ production upon KYNA treatment in five different concentration (0,005–50 µM) (**k**) BRAF V600E cell lines transiently transfected with expression plasmids encoding BRAF wt and corresponding lysates were subjected to immunoblotting with the indicated antibodies (**l,m**) the proliferation of CD4+ T-cells (three different donors) and IFNγ production in culture with transfected cell lines (SK.mel-28 and DFB) on day five by flowcytometery. Graphs show individual data, and horizontal lines show mean ± s.e.m. ****P ≤ 0.0001, ***P ≤ 0.0005 **P ≤ 0.001, *P ≤ 0.05, the Tukey multiple comparison procedure was used to determine significance and adjusted P values for the differences in group means can be seen in Table S6, n > three biological replicates cultured with four different melanoma cell lines.

In order to investigate the role of the KP metabolites in district CD4+ T-cell behavior in co-culture set-up, CD4+ T-cell proliferation and KP metabolites in each culture were evaluated separately. In the closer look, we noticed CD4+ T-cells were less proliferative in the presence of BRAF wt MCLs compared with V600E MCLs (Fig. 4a). Despite Tryptophan depletion, no significant differences were detected in KYN production among different MCLs (Supplementary Fig. 1b, Fig. 4b). Therefore, to convey the anti-proliferative role of TRP depletion on CD4+ T-cell, a rescue experiment was conducted by adding TRP on co-culture set-up. Subsequently, CD4+ T-cell proliferation was measured, which revealed that additional TRP did not restore CD4+ T-cell proliferation (Supplementary Fig. 3a). Thus, it is possible that TRP depletion itself may not have any anti-proliferative effect on CD4+ T-cells. On the other hand, KYNA production was higher in V600 wt MCLs (BE) co-cultured with CD4+ T-cells compared with V600E cell lines (A375, SK-MEL-28, and DFB). Interestingly, the PD-L1 and AHR mRNA expression were higher in BRAF wt MCLs cultured with CD4+ T-cell compared with V600E MCLs (Fig. 4d,f). However, AHR mRNA expression was also higher in CD4+ T-cells upon co-culture with V600E MCLs (Fig. 4e). This observation suggests that functional implications of elevated KYNA and CD4+ T-cell exhaustion may be regulated by PD-L1 and AHR expression. We, therefore, further evaluated the effect of KYNA production on CD4+ T-cell proliferation.

To investigate the potential role of KYNA in CD4+ T-cell exhaustion, CD4+ T-cell were treated with different concentrations of KYNA (0,005–50 µM). The proliferation of CD4+ T-cell and IFNγ production measured on day five and reveals that the presence of KYNA can revoke the CD4+ T-cell proliferation and IFNγ production (Fig. 4g–j). Figure 4g show representative images of gating strategy (left panel), IFNγ production (right upper panel) and CD4+ T-cell proliferation (right lower panel) in different concentrations of KYNA. Cell toxic concentration of KYNA was defined using viability assay by flow cytometry and concentrations of KYNA lower than 0.05 µM did not show any toxicity (Fig. 4h) and concentration 0.05 μM effectively inhibited CD4+ T-cell proliferation and IFNγ production (Fig. 4i,j).

Moreover, to verify the association of BRAF wt status on CD4+ T-cell exhaustion, V600E cell lines (SK-MEL-28 and DFB) were transiently transfected with expression vector encoding BRAF wild-type and co-cultured with CD4+ T-cell for five days (Fig. 4k). The proliferation of CD4+ T-cell and IFNγ production was measured on day five which supports that BRAF wt phenotype has a profound effect on CD4+ T-cell exhaustion when compared to BRAF V600E (Fig. 4k–m).

### IFNγ triggers KYN and KYNA production but not 3-HK of melanoma tumours

**Figure 5:**
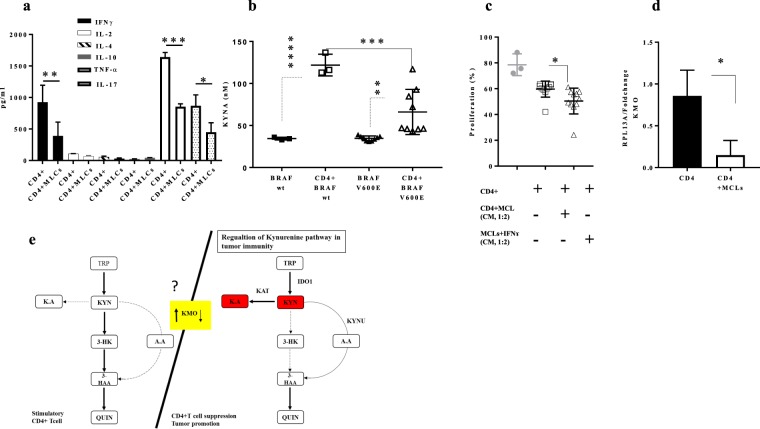
The IFNγ–triggered KYN and KYNA induction in CD4+ and MCLs co-culture. (**a**) IFNγ, IL-2, IL-4, IL-10, TNFα and IL-17 protein expression measured by multiplex Luminex system in the culture supernatants of pre-activated CD4+ only and pre-activated CD4+ cultured with MCLs (**b**) KYNA production measured by LC-MS/MS on MCLs treated with recombinant human (rh) IFNγ (50 ng/ml) in V600 wt and V600E for 48 hours (**c**) functional assessment of the CD4+-MLCs derived cultured medium on CD4+ T-cell proliferation after 5 days (**d**) KMO mRNA expression from educated CD4+ T-cells (**e**) Regulation of kynurenine pathway in tumour immunity. (Left panel) KMO up-regulation during CD4+ T-cell activation results in a shift in kynurenine metabolism that increases the production of QA. (Right panel) KMO dysfunction as a consequence of exposure to melanoma favours the production of KA and Kats activities. Graphs show individual data, and horizontal lines show mean ± s.e.m. ****P ≤ 0.0001, ***P ≤ 0.0005, **P ≤ 0.001, *P ≤ 0.05 by independent samples t-test (two-sided) and the Tukey multiple comparison procedure was used to determine significance, n = four different melanoma cell lines.

Due to the close link between KP activation and inflammatory stimuli, a Type 1 T helper (Th1) cells cytokine profile including IFNγ, TNFα, IL-2 and Type 2 T helper (Th2) cytokine profile including IL-4, IL-2 and IL-10 were performed on culture supernatants and measured using a multiplex Luminex assay (Fig. 5a). IFNγ and TNFα were the central cytokines which were continuously secreted upon CD4+CD25− T-cell activation in our set-up. IL-2 production, which is a critical cytokine for T-cells survival and maintenance, was not changed in the co-culture set up (Fig. 5a). In order to investigate the role of IFNγ and TNFα on KP activation, MCLs were treated with recombinant human IFNγ (50 ng/ml) and TNFα (10 ng/ml) for 48 hours. We did not observe any changes after TNFα treatment in IDO1 expression or KYN production. Furthermore, IFNγ treatment was able to induce the KYN, KYNA, and AA production while the 3-HK production stayed very low (Supplementary Fig. 3b–e). Moreover, CD4+ T-cells were less proliferative in culture with conditioned medium (CM) derived from IFNγ treated MCLs compared with CM derived from MCLS+CD4+ T-cell co-culture (Fig. 5c). The elevated KYNA may explain this in IFNγ treated MCLs compared with CD4+ T-cell treated MCLs. Furthermore, KYNA production in BRAF wt was higher than BRAF V600E MCLs (Fig. 5b). Interestingly, lower KMO mRNA expression was seen in CD4+ T-cells upon co-culture with MCLs (Fig. 5d), suggesting that KMO may play a role in determining the levels of KYNA and may also be an exciting candidate regulating the kynurenine pathway in tumour immunity (Fig. 5e). Additionally, basal and IFNγ-driven mRNA expression of KP enzymes including IDO1, IDO2, TDO2, and KMO in MCLs were measured, IDO2, and TDO2 expression were under detection level (Supplementary Fig. 3f,g). On the other hand, after IFNγ treatment, KYN production was induced, but there was no significant difference between KYN productions in MCLs (Supplementary Fig. 3b). This result suggests that induction of CD4+ T-cell exhaustion in co-culture set-up may depend on elevated KYNA.

### Profiling the correlation networks for CD4+ T-cell and KP-related genes in BRAF V600E compared with BRAF wt SKCM

**Figure 6:**
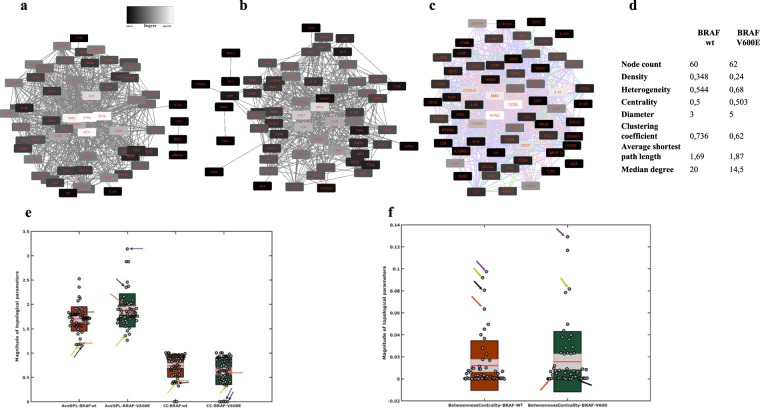
Correlation-based network analysis of CD4+ T-cells and KP metabolites-related genes in V600E and BRAF wt SKCM (**a**) correlation networks between CD4+ T-cells and KP metabolites-related signature genes in BRAF V600E SKCM and in (**b**) BRAF wt SKCM (**c**) DyNet visualization of union correlation network of CD4+ T-cells and KP metabolites-related signature genes; the pink edges are present only in the case of BRAF wt; green edges are present only in BRAF V600E; light purple edges are present in both. DyNet highlights the most rewired nodes. More red nodes with higher varying edge connections via the betweenness score (**d–f**); arrows indicate different genes, black; KYNU, orange; KMO, olive; IDO1, blue; TGFβ1, purple; IDO2. CC; average clustering coefficient, AveSPL; average shortest path length.

Our results suggest the possible relation between the mutation status of the MCLs and KP metabolites in district CD4+ T-cell behaviour in co-culture set-up. We, therefore, evaluated whether KP retains dissimilar anti-tumoural CD4+ T-cell immune response in BRAF V600E compared with BRAF wt SKCM in a broader cohort or not. We reconstructed a network by thresholding Spearman correlation among CD4+ T-cells and KP-related genes for each group (BRAF V600E & BRAF wt). The signature genes associated with the CD4+ T-cells and KP were extracted from PubMed (Table S5), using patient data available in the TCGA database (SKCM, 2016). We then used these Spearman correlation values (absolute value of Spearman correlation ≥0.5) between CD4+ T-cell representative genes and metabolites of interest to reconstruct networks (Fig. 6a,b). To visualize the difference between these two networks, we then merged both networks using DyNet. Differences between the two networks based on variation in node betweenness were highlighted in the merged network (Fig. 6c). Comparing the structure of these networks revealed interesting structural dissimilarities between BRAF V600E and BRAF wt networks. First, we characterized the network’s topological parameters [clustering coefficient, network centralization, network density, network diameter, network heterogeneity, and network radius (Fig. 6d).

The number of interactions in the BRAF V600E network was less than BRAF wt, while both centrality and heterogeneity of the network were increasing (Fig. 6d). Nodes in the BRAF wt network tended to have a higher degree than those in the BRAF V600E network. The total number of edges of the BRAF wt network (n = 616) was significantly higher than the BRAF V600E network (n = 444). The median degree value of 20 in the BRAF wt network decreased to 14.5 in the BRAF V600E network. The magnitude of the average clustering coefficient went down from 0.73 for the BRAF wt network to 0.62 in the BRAF V600E network, indicating greater clustering tendency of the BRAF wt network. All of these changes indicated distinct properties of the BRAF V600E and BRAF wt networks). KYNU, IDO1/2, and KMO had the highest degree and were among the top-ranked nodes in terms of other topological properties in the BRAF wt network (Table S6). The topological profile of KYNU and KMO in BRAFV600E-network changed utterly. They, lost their central role in the network while IDO1 and IDO2 almost kept the same topological profiles (Fig. 6e,f). Interestingly, the EdgeBetweenness of CD86-KYNU increased from almost the bottom-ranked EdgeBetweenness (411) in BRAF wt to top-ranked (6) in BRAF V600E (Table S6) which suggests that perturbing (removing) these two nodes can have a considerable effect on the BRAF V600E network. These remarks support our previous observation in co-culture set-up and suggest that KMO and KYNU may play a role in district CD4+ T-cell behaviour in tumour immunity.

Collectively, KP and CD4+ T-cell interactions in the BRAF wt and V600E networks suggest that kynurenine metabolism may have an essential role in the dysregulation of the anti-tumoural CD4+ T-cell immune response.

## Discussion

In the present study, we established an *in vitro* co-culture model consisting of CD4+CD25- T-cells obtained from healthy volunteers in culture with four different MCLs (BRAF wt and BRAF V600E) to investigate the implication of kynurenine pathway alteration on CD4+ T-cell function. We found that the enzymatic activity of IDO1 is significantly up-regulated in MCLs when exposed to IFNγ producing CD4+ T-cells. Supporting our results, the analysis of the SKCM-TCGA database revealed a robust correlation between KP enzymes and inhibitory checkpoints (Fig. 1b, Supplementary Fig. 5b). Furthermore, higher KYN and KYNA production resulted in an anergic phenotype in CD4+ T-cells, with defects in proliferation.

Additionally, by using correlation networks between kynurenine pathway metabolites and CD4+ T-cell parameters, we identified KMO and KYNU as central nodes in network diagrams built around data from BRAF V600E and BRAF wt in SKCM -TCGA data. These findings not only support the immunomodulatory potential of the kynurenine pathway in the tumour microenvironment but also display KMO and KYNU alteration in the kynurenine pathway that regulates the immune function of CD4+ T-cells. Besides, the full spectrum of TRP metabolites towards KP, upon exposure of CD4+ T-cells to MCLs, showed that Th1-type cytokines IFNγ led to a higher production of KYN and KYNA compared with 3-HK.

On the other hand, KYNA production was notably higher in BRAF wt MCLs co-cultured with CD4+ T-cells compared with BRAF V600E cell lines, although no significant differences were detected in KYN production among different MCLs. Furthermore, treating MCLs with IFNγ alone led to a higher production of KYNA in BRAF wt MCL compared with BRAF V600E MCLs. In addition, CD4+ T-cells were less proliferative in culture with VE600wt MCLs compared with VE600E MCLs, which may reflect the critical role of elevated KYNA on the induction of CD4+ T-cell exhaustion in co-culture set-up. Additionally, the lower mRNA KMO expression in CD4+ T-cells and elevated level of KYNA upon co-culture may propose KMO as an enzyme that is crucial for determining the levels of KYNA, which has also been reported elsewhere^34^.

Our data also support the idea that Epacadostat (INCB024360), a potent inhibitor of IDO1 with direct reactivation of CD4+ T-cells *in vitro*^35^, mediates the proliferation of CD4+ T-cells and abrogates CTLA4 expression on CD4+ T-cells and PD-L1 expression in MCLs (Supplementary Fig. 2f). Recent studies with a combination of INCB024360 and the PD-1 blockade (pembrolizumab) in patients with advanced melanoma did not show any significant improvement compared with the single PD-1 blockade, which supports the clinical relevance of our studies and indicates that IDO1 inhibition alone will not enhance the effect of immune checkpoint blockade^7,36^.

Altogether, our data suggest that, in addition to IDO1, other kynurenine metabolites may regulate the CD4+ T-cells immunity. Moreover, reduction of KMO expression after its exposure to MCLs may serve as an exciting candidate in regulating the kynurenine pathway in tumour immunity (Fig. 5e) and opens up the possibility that disrupting the metabolism of kynurenines may be used to design novel and more effective treatment strategies. However, more research is still required for a better understanding of the pathogenesis of KP metabolites and their intra- and interconnectivity for a great variety of diseases and disorders.

## Methods

### Antibodies and reagents

Recombinant IFNγ (cat. 285-IF-100) and 10 µg/mL neutralizing antibodies against IFNγ (cat. AB-285-NA) and anti-mouse IgG1 (clone11711) were purchased from R&D system (McKinley Place NE). INCB024360 (CAS 914471-09-3, 98% pure) was purchased from MedChem Express. L-Tryptophan and KYNA were purchased from Sigma-Aldrich Sweden. For vector transfection, Lipofectamin 3000 was purchased from ThermoFisher Scientific (#L3000008). Wild type BRAF plasmid was a gift from Dustin Maly (Addgene plasmid # 40775; http://n2t.net/addgene:40775; RRID: Addgene_40775). Empty vector pcDNA3.1 and GenElute Plasmid Miniprep Kit (PLN70) were purchased from Invitrogen and Sigma Aldrich, respectively. Primary antibodies, Raf-B (SC-5284) and β-Actin (5125) were purchased from Santa Cruz Technology and Cell Signalling Technology, respectively. Detailed information about the purchased antibodies is provided in Table S1.

### CD4+ and cancer cell co-culture assays, transfection, and treatment

Buffy coat samples from healthy donors were acquired from Karolinska University Hospital blood bank, Sweden.

Lymphocytes were isolated from fresh buffy coats by using density gradient centrifugation with Ficoll-Paque Plus (GE Healthcare). CD4+ T-cell subsets were then isolated from PBMC’s negative selection, and the CD25+ fraction was additionally depleted with CD25-specific MACS beads following the manufacturer’s instructions (Miltenyi Biotec). CD4+ T-cell subsets were pre-activated overnight with LEAF™ purified anti-human CD3 antibody (2 mg/ml) and CD28 antibody (1 ug/ml). Co-culture models used for investigating the cellular interactions *in vitro* (Supplementary Fig. S1b), MCLs (BE, DFB, A375 and SK-MEL-28), were incubated for 24 hours at a concentration of 1.0 × 10^4^ cells/ml with 1 × 10^6^ cells⁄ml of activated CD4+ T-cells on the following day and subsequently co-cultured for up to 5 days, with and without aIFNγ and blocking antibody. The two populations were then separated by labeling the CD4+ T-cells against the tumour, using Dynal® positive isolation kits. The RNA was quantified via TaqMan real-time PCR (Applied Biosystems), and the total amount extracted using an RNeasy Mini kit (Qiagen). Our experimental procedure involved the use of an RNA isolation system, a one-step real-time RT-PCR assay, and a SuperScript VILO cDNA Synthesis Kit (Invitrogen). RPL13A (Ribosomal Protein L13a1) was used as a reference gene. Empty vector pcDNA3.1 and wild-type BRAF expression plasmid were prepared by GenElute Plasmid Miniprep Kit. Immunoblots were performed on whole protein lysates from SK-MEL-28 and DFB cell lines transfected for 72 h with vectors expressing wild type BRAF or empty control vectors following standard protocol from Lipofectamin 3000. The images are not cropped or adjusted in any manner. The exposure time for the figure is three seconds for BRAF and β-actin (Supplementary Fig. 6a,b).

### Flow cytometry

Cell surface staining protocol with anti-CD45RA-FTIC, CD4-PerCP/Cy5.5, CD25-PE, CD8A-APC, CD14-PE, and CD19-APC (Table S1) was performed to verify the purity of CD4+ T-cells. Viability and proliferation stainings were performed using a fixable viability dye, eFluor 780, and a cell proliferation dye, carboxyfluorescein diacetate succinimidyl ester (CFSE), respectively. Before all intracellular staining, cells were restimulated for 4 hours using ten ng/ml of phorbol 12-myristate 13-acetate (PMA, Sigma) and 375 ng/ml of ionomycin (Iono), in the presence of GolgiPlug protein transport inhibitor containing Brefeldin A (BFA; BD Biosciences). The following human antibodies were used: CD152-APC, CD279-PE, CD274- BV421, FOXP3-APC, IDO1- Alexa flour 700, mouse, IgG2Isotype Control-APC, mouse, IgG2b-PE, mouse IgG1-Alexa flour 700 (Table S1). A CyAn ADP 9-Colour Analyser (Beckman Coulter) was used for flow cytometry recording, and CyAn software (Summit) was used for creating the compensation settings using single-stained compensation beads (BD CompBeads, BD Biosciences). Data of flow cytometry were analysed using a FlowJo® software (Tree Star).

### Cytokine production

Levels of the cytokines IFNγ, tumour necrosis factor alpha (TNFα), IL-10, IL-17, IL-2, and IL-4 in culture medium were determined using a multiplex bead array assay. All the reagents were purchased from R&D Systems. Individual bead sets (Luminex) were coupled to cytokine-specific capture antibodies following the manufacturer’s instructions. Data were recorded on the Bio-Plex-200 platform and analysed using the Bioplex Manager® software (version 6.1; Bio-Rad) with a 5-parameter logistic regression algorithm.

### HPLC/MS

TRP and KYN were measured by HPLC/MS. HPLC/MS was performed using a medium derived from the *in vitro* co-culture of CD4+ CD25− T-cells from three healthy donors and human MCLs. Detailed characteristics of MCLs can be found in Table S2. The KYN/TRP ratio was used as a surrogate indicator of IDO1 activity. Levels of TRP and KP metabolites in cell culture media were analysed by isocratic liquid chromatography with ultraviolet detection as described in previous work^37^.

### HILIC–MS/MS metabolomics

Kynurenines were measured by HILIC mass spectrometry using sample processing, and analyses were performed as previously described^35^. Briefly, the culture supernatants of melanoma cell lines alone, CD4+ T-cells alone and MCLs- CD4+ T-cells co-cultures were removed from storage (−80 °C) and thawed on ice. 50 μl of cell media were diluted using a 4-volume ratio of HPLC grade acetonitrile (Rathburn Chemicals). Samples were vortexed for 5 seconds, left to stand on ice for 10 minutes and then centrifuged (Eppendorf Centrifuge 5430 R) at 15000 rcf for 10 minutes at 4 °C. The supernatant (150 μl) was transferred to a Chromacol clear high-recovery chromatography glass vials with a sealed insert (03-FISV Thermo Fischer) and capped with a pre-slit PFTE caps (03-FISV Thermo Fischer 9-SC(B)-ST1X).

Samples were analysed on a Thermo Ultimate 3000 UHPLC and Thermo Q-Exactive Orbitrap mass spectrometer. 10 μl of the sample was injected into a Merck-Sequant ZIC-HILIC column (150 × 4.6 mm, 5 μm particle size) fitted with a Merck Sequant ZIC-HILIC guard column (20 × 2.1 mm). A 30 min gradient (0.4 ml/min flow rate, 23 °C column oven) using 0.1% formic acid in HPLC water (mobile phase A) (Milli-Q, Millipore) and 0.1% formic acid in HPLC acetonitrile (mobile phase B) (Rathburn Chemicals) was applied. The gradient started at 80% B reducing to 20% B after 18 min and stabilized at 80% A for 4 minutes and then returned to initial conditions followed by an 8-minute column re-equilibration. Mass spectrometry data were acquired (full scan mode) in both positive and negative ionization modes (an independent run for each polarity), using a mass range of 75 and 1000 m/z with a resolution of 70,000 at 400 m/z. In positive mode, the spray voltage was 4.0 kV with a capillary temperature of 350oC, a sheath gas flow of 30 and an auxiliary gas flow of 10 (arbitrary units by the vendor). In the negative mode, the spray voltage was 3.6 kV with a capillary temperature of 350oC, a sheath gas flow of 30 and an auxiliary gas flow of 12 (arbitrary units by the vendor). Samples were randomized across each batch to prevent potential confounding signal drift.

### Analysis of the cancer genome atlas (TCGA) data

Level 3 gene expression data of mRNA sequencing on skin cutaneous melanoma (SKCM) were acquired from TCGA that was processed by the Broad Institute’s pipeline (Firehose run “28 January 2016”: 10.7908/C11G0KM9) (Supplementary Table S4). Our analysis run was based on the 2016_01_28 data run and included 368 metastatic SKCM samples. T-cell signature genes shown in previous studies^34^ were used for clustering metastatic SKCM samples to T-cell signature high and low groups. For cluster analysis, we applied Ward’s method using the Euclidean distance, and a correlation coefficient was used for genes with the same method. Correlation coefficients for CD4 or IDO1 with genes of interest were calculated using the Spearman correlation coefficient.

### Network analysis

In the correlation network analysis, a node represents a kynurenine pathway metabolite or CD4+ T-cell related genes, and an edge is defined by statistically significant correlations between genes in two groups. These values (absolute value of Spearman correlation ≥0.5; this is the highest level of correlation where the network is not fragmented) were used to reconstruct networks in BRAF V600E and BRAF wt SKCM groups^38^. A network analysis was carried out using a cystoscope (3.4.0).

### Statistical analysis

The cycle threshold (Ct) value for the genes was determined using StepOneTM Real-Time PCR software v2.0 (Applied Biosystems), and gene expression was normalized to the internal control RPL13A. The relative gene expression compared to the control group was calculated as 2^−ΔΔCt^. Flow cytometry data were analysed using the FlowJo® software (Tree Star). Statistical significance was determined using the Student’s *t*-test and was considered significant if *P* < 0.05.

### Ethical approval and informed consent

The studies described in the manuscripts involved no human participants.

### Consent for publication

This manuscript does not contains any individual person’s data in any form.

### Cell lines authentication

Melanoma cell lines BE, DFB, A375 and SK-MEL-28 were cultured in RPMI 1640 medium with 10% heat-inactivated FBS and penicillin/streptomycin. Low-passage cell lines were received in 2013, frozen in ampoules and thawed for experiments; no changes in phenotype or morphology have been documented (7 passages). Furthermore, mycoplasma testing was routinely performed (LookOut Mycoplasma PCR Detection kit, Sigma Aldrich, MP0035), verifying that they were mycoplasma free (Supplementary Fig. S5).

## Supplementary information

Supplementary materials

## Data Availability

All data generated or analysed during this study are included in this published article.
