# Supplementary material for: Exhaustion of CD4+ T-cells mediated by the Kynurenine Pathway in Melanoma
Source: Sci Rep. 2019 Aug 21;9:12150. doi: 10.1038/s41598-019-48635-x (PMC6704156; doi:10.1038/s41598-019-48635-x)
Supplement: Supplementary file 1 — Supplementary materials [file 41598_2019_48635_MOESM1_ESM.pdf]

Title

Exhaustion of CD4+ T-cells mediated by the Kynurenine Pathway in Melanoma

Author list and affiliation

Soudabeh Rad Pour[1*], Hiromasa Morikawa¶[7], Narsis A. Kiani¶[1,2], Muyi Yang¶[3], Alireza Azimi[8], Gowhar Shafi[6], Mingmei Shang[1], Roland Baumgartner[4], Daniel FJ Ketelhuth[4], Muhammad Anas Kamleh[5], Craig E. Wheelock[5], Andreas Lundqvist[3], Johan Hansson[3], Jesper Tegnér[1,7].

[1]Unit of Computational Medicine, Department of Medicine, Centre for Molecular Medicine, Karolinska Institute, SE-171 76, Stockholm, Sweden

[2]Algorithmic Dynamics Lab, Unit of Computational Medicine, Department of Medicine Solna, Centre for Molecular Medicine, Karolinska Institute and SciLifeLab, SE-171 77 Stockholm, Sweden

[3]Department of Oncology-Pathology, Cancer Centre Karolinska, Karolinska University Hospital, Stockholm, Sweden

[4]Experimental Cardiovascular Research Group, Cardiovascular Medicine Unit, Centre for Molecular Medicine, Department of Medicine, Karolinska Institute, Karolinska University Hospital, SE-171 76 Stockholm, Sweden

[5]Division of Physiological Chemistry II, Department of Medical Biochemistry and Biophysics, Karolinska Institute, SE-171 77 Stockholm, Sweden

[6]Department of Genomics and Bioinformatics, Positive Bioscience, Mumbai -400 002, India

[7]Biological and Environmental Sciences and Engineering Division (BESE), Computer, Electrical, and Mathematical Sciences and Engineering Division (CEMSE), King Abdullah University of Science and Technology (KAUST), Thuwal 23955–6900, Kingdom of Saudi Arabia

[8]Department of Immunology, Genetics & Pathology, Science for Life Laboratory, Uppsala University, Uppsala, Sweden.

¶ Equal contribution of authors

* Corresponding author

Corresponding author

Soudabeh Rad Pour

Unit of Computational Medicine

Centre for Molecular Medicine

L8:05 Karolinska University Hospital

SE-171 76 Solna, Sweden

Phone: +46704040672

Email: soudabeh.rad-pour@ki.se

Table S1 Antibodies for analysis of CD4+ T cells and melanoma cells

Name	color	Company	Catalog Number	comments
**CD152**	APC	Miltenyi	130-097-682	BNI3
**mouse, IgG2**	APC	Miltenyi	130-098-850	130-098-850
**CD279**	PE	Miltenyi	130-096-164	PD1.3.1.3
**mouse, IgG2b**	PE	Miltenyi	130-098-875	IS6-11E5.11
**CD3**	PE-Vio770	Miltenyi	130-098-150	BW264/5
**CD274**	BV421	BD Biosciences	563738	MIH1
**CD3**	PerCP	BD Biosciences	340663	SK7
**CD4**	FITC	eBiosciense	11-0049-42	RPA-T4
**CD4+**	BUV310	BD Biosciences	564652	SK3
**FoxP3**	APC	eBiosciense	17-4777-42	236A/E7
**IDO1**	Alexa flour	R&D	IC6030G	700838
**Mouse IgG1**	Alexa flour	R&D	IC002G	11711
**IFNG**	PE-vio770	Miltenyi	130-096-752	45-15
**mouse, IgG1**	PE-Vio770	Miltenyi	130-099-062	X-56

Table S2 Characteristics of melanoma cell lines included in this study

**Cell lines**	**BRAF**	**NRAS**	**TP53**	**CDK4**	**CDKN2A**	**biological source**
BE	WT	+	+	WT	NA	Sentinel Lymph Node
SK-mel-2	WT	+	+	NA	NA	Skin
DFB	+	WT	+	WT	NA	Sentinel Lymph Node
A375	+	WT	WT	NA	+	Skin
SK-mel-28	+	WT	+	NA	+	Skin

Table S3 Spearman correlation coefficient analyses on kynurenine pathway-related and T-cell status-related genes[1]

	IDO1	IDO2	TDO2	KMO	KYNU	CCBL1	CCBL2	GOT2	AADAT	QPRT
**CD2**	0,87	0,77	0,44	0,64	0,58	0,11	0,06	-0,23	-0,26	-0,12
**CD3D**	0,84	0,76	0,43	0,62	0,55	0,15	0,07	-0,20	-0,28	-0,08
**CD3E**	0,82	0,73	0,41	0,61	0,56	0,16	0,03	-0,18	-0,31	-0,05
**CD3G**	0,82	0,71	0,42	0,62	0,54	0,06	0,09	-0,24	-0,23	-0,13
**CD8A**	0,86	0,69	0,36	0,61	0,53	0,10	0,04	-0,25	-0,20	-0,09
**SIRPG**	0,84	0,73	0,37	0,61	0,55	0,16	0,05	-0,19	-0,31	-0,04
**TIGIT**	0,87	0,74	0,43	0,64	0,56	0,10	0,07	-0,23	-0,25	-0,11
**GZMK**	0,82	0,71	0,44	0,62	0,55	0,09	0,06	-0,25	-0,23	-0,14
**ITK**	0,78	0,71	0,46	0,58	0,59	0,14	0,09	-0,16	-0,25	-0,08
**SH2D1A**	0,84	0,76	0,46	0,65	0,55	0,08	0,13	-0,28	-0,19	-0,18
**CD247**	0,82	0,76	0,44	0,62	0,56	0,17	0,06	-0,22	-0,26	-0,10
**PRF1**	0,80	0,65	0,38	0,58	0,54	0,15	0,01	-0,19	-0,25	-0,01
**NKG7**	0,83	0,68	0,36	0,59	0,53	0,15	-0,01	-0,19	-0,24	-0,03
**IL2RB**	0,81	0,74	0,46	0,59	0,56	0,12	0,00	-0,22	-0,24	-0,08
**SH2D2A**	0,62	0,64	0,28	0,43	0,48	0,16	-0,04	0,03	-0,22	0,04
**KLRK1**	0,77	0,71	0,42	0,60	0,48	0,12	0,08	-0,31	-0,11	-0,17
**ZAP70**	0,74	0,73	0,41	0,56	0,44	0,19	0,10	-0,19	-0,26	0,03
**CD7**	0,80	0,72	0,37	0,58	0,56	0,20	0,01	-0,17	-0,28	0,00
**CST7**	0,72	0,62	0,37	0,56	0,60	0,14	-0,02	-0,15	-0,25	-0,01
**LAT**	0,57	0,63	0,40	0,45	0,51	0,23	-0,10	-0,09	-0,11	-0,11
**PYHIN1**	0,75	0,60	0,32	0,57	0,43	0,05	0,18	-0,32	-0,25	-0,06
**SLA2**	0,85	0,76	0,43	0,62	0,56	0,15	0,06	-0,23	-0,26	-0,08
**STAT4**	0,78	0,78	0,52	0,63	0,52	0,07	0,15	-0,31	-0,14	-0,20
**CD6**	0,78	0,74	0,42	0,57	0,54	0,18	0,00	-0,15	-0,28	-0,05
**CCL5**	0,85	0,68	0,38	0,61	0,56	0,13	-0,01	-0,20	-0,25	-0,03
**CD96**	0,79	0,72	0,42	0,61	0,59	0,11	0,04	-0,26	-0,17	-0,20
**TC2N**	0,46	0,50	0,30	0,41	0,34	0,04	0,04	-0,20	-0,06	-0,26
**FYN**	0,34	0,34	0,14	0,18	0,23	0,01	0,04	0,01	-0,18	0,00
**LCK**	0,83	0,75	0,43	0,61	0,57	0,16	0,04	-0,20	-0,29	-0,07
**TCF7**	0,30	0,42	0,22	0,27	0,32	0,30	-0,14	-0,10	-0,14	0,02
**TOX**	0,70	0,63	0,37	0,45	0,42	0,14	0,04	-0,24	-0,18	-0,16
**IL32**	0,80	0,71	0,45	0,59	0,59	0,19	-0,02	-0,15	-0,27	-0,07
**SPOCK2**	0,78	0,72	0,40	0,58	0,54	0,14	0,01	-0,17	-0,32	-0,03
**SKAP1**	0,55	0,49	0,30	0,56	0,41	0,13	-0,03	-0,12	-0,04	-0,17
**CD28**	0,62	0,62	0,49	0,59	0,45	0,06	0,08	-0,29	-0,17	-0,18
**CBLB**	0,23	0,26	0,32	0,28	0,34	-0,02	0,09	-0,16	-0,05	-0,16
**APOBEC3G**	0,69	0,58	0,35	0,56	0,48	0,07	-0,07	-0,23	-0,04	-0,21
**PRDM1**	0,72	0,65	0,51	0,55	0,57	0,08	0,03	-0,27	-0,06	-0,23

Table S4 Inflammatory mediators of enzyme activities along the kynurenine pathway [2,3,4]

Enzyme	Abbreviations	Inflammatory mediators	
		Up-regulation	Down-regulation
IDO1	indoleamine 2,3-dioxygenase 1	IFNs, TNF-α[5], IL-1β[6], IFN-gamma[7]	
IDO2	indoleamine 2,3-dioxygenase 1	IFNs, TNF-α, IL-1β, IFN-gamma	
TDO2	tryptophan 2,3-dioxygenase	IFNs, TNF-α, IL-1β, IFN-gamma	
KMO	kynurenine 3-monooxygenase	IFN-gamma, IL-1β	
KYNU	kynureninase	IFN-gamma, IL-1β	
KAT	kynurenine aminotransferase		IL-1beta, IFN-gamma
3-HAO	3-hydroxy-anthranilate		
ACMSD	α-amino-β-carboxymuconate-ε-semialdehyde decarboxylase		IFN-gamma

Table S5 The signature genes associated with the CD4+ T cells and KP metabolites

CD4 T cell markers
ADA
APC
BCL2
BLM
CCL3
CCR1
CCR2
CCR3
CCR4
CCR5
CCR6
CCR8
CD1D
CD2
CD27
CD274
CD276
CD28
CD3D
CD3E

CD3G
CD4
CD40
CD40LG
CD47
CD5
CD7
CD80
CD86
CD8A
CD8B
CSF2
CXCR3
CXCR4
DPP4
EGR1
FOXP3
GATA3
ICAM1
ICOSLG
IFNB1
IFNG
IL10
IL12A
IL12B
IL12RB1
IL12RB2
IL13
IL15
IL17A
IL18
IL18R1
IL18R1
IL1B
IL2
IL23A
IL2RA
IL3
IL4
IL4R
IL5
IL6
IRF4
LAG3
LCK

MAP3K7
MICB
NCK1
NOD2
PTPRC
RIPK2
RORC
SLC11A1
SOCS1
STAT3
STAT4
STAT6
TBX21
TGFB1
TLR2
TLR4
TLR6
TLR9
TNFSF14
VAV1

References

1. Tirosh I, Izar B, Prakadan SM, et al. Dissecting the multicellular ecosystem of metastatic melanoma by single-cell RNA-seq. 2016;16. doi:10.1126/science.aad0501.

2. Campbell BM, Charych E, Lee AW, Möller T. Kynurenines in CNS disease: Regulation by inflammatory cytokines. *Front Neurosci*. 2014;8(8 FEB):1-22. doi:10.3389/fnins.2014.00012.

3. Amori L, Guidetti P, Pellicciari R, Kajii Y, Schwarcz R. On the relationship between the two branches of the kynurenine pathway in the rat brain in vivo. *J Neurochem*. 2009;109(2):316-325. doi:10.1111/j.1471-4159.2009.05893.x.

4. Adams S, Teo C, McDonald KL, et al. Involvement of the kynurenine pathway in human glioma pathophysiology. *PLoS One*. 2014;9(11):1-28. doi:10.1371/journal.pone.0112945.

5. Chon SY, Hassanain HH, Gupta SL. Cooperative Role of Interferon Regulatory Factor 1 and p91 (STAT1) Response Elements in Interferon- -inducible Expression of Human Indoleamine 2,3-Dioxygenase Gene. *J Biol Chem*. 1996;271(29):17247-17252. doi:10.1074/jbc.271.29.17247.

6. Babcock TA, Carlin JM. Transcriptional Activation of Indoleamine Dioxygenase By Interleukin 1 and Tumor Necrosis Factor A in Interferon-Treated Epithelial Cells. *Cytokine*. 2000;12(6):588-594. doi:10.1006/cyto.1999.0661.

7. Pallotta MT, Orabona C, Volpi C, et al. Indoleamine 2,3-dioxygenase is a signaling protein in long-term tolerance by dendritic cells. *Nat Immunol*. 2011;12(9):870-878. doi:10.1038/ni.2077.

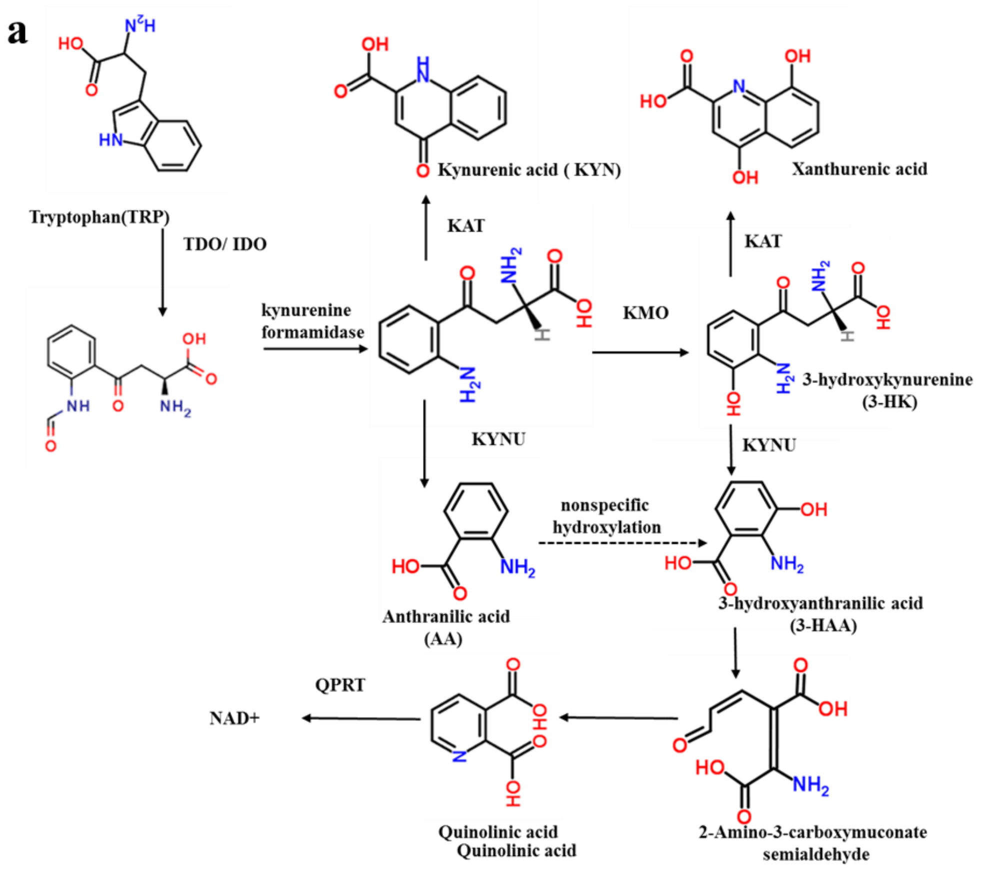

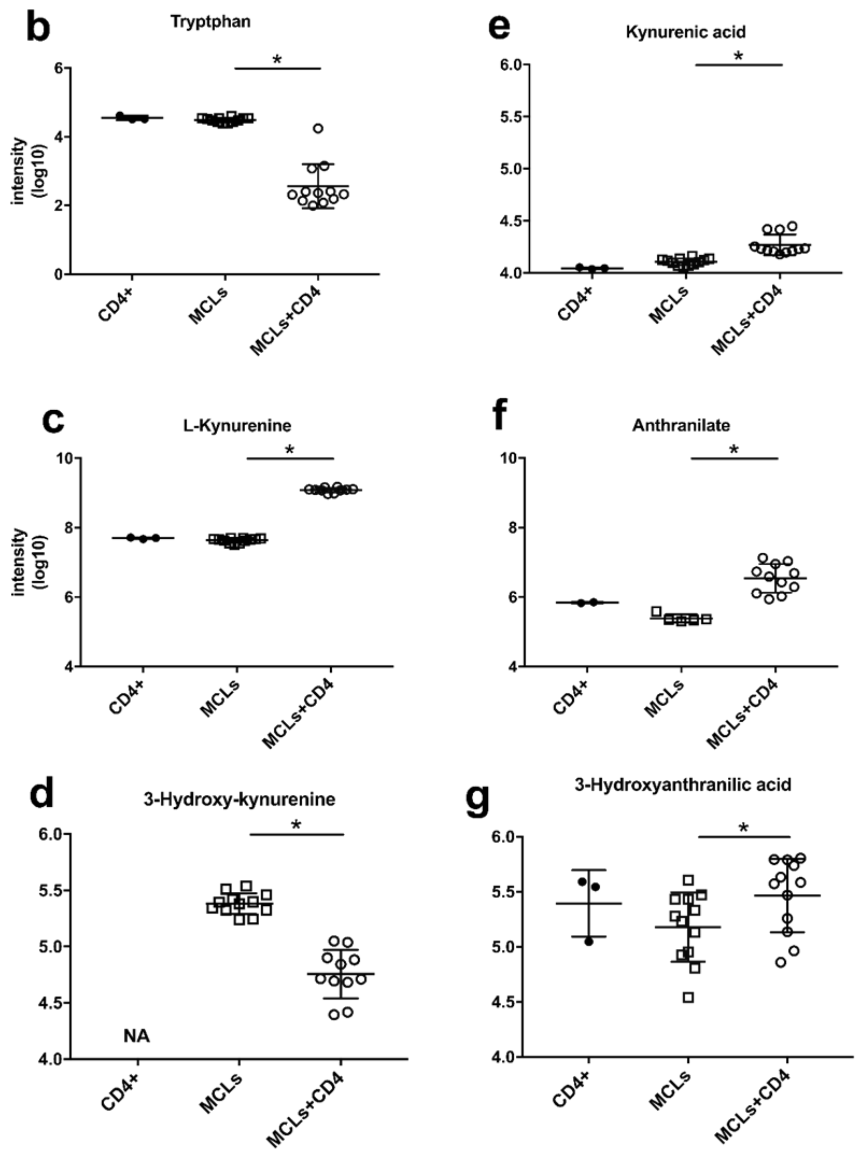

Figure S1. Concentrations of kynurenine metabolites from culture-derived supernatant measured by LC-MS/MS. **a**, Scheme of the kynurenine pathway. **b**, TRP. **c**, KYN. **d**, 3-HK. E, KYNA. **f**, 3-HA and **g**, AA. Graphs display individual data points, and horizontal lines display mean ± s.e.m. *$P \leq 0.05$ by independent samples t test (two-sided), n = three biological replicates of experiments with four different melanoma cell lines.

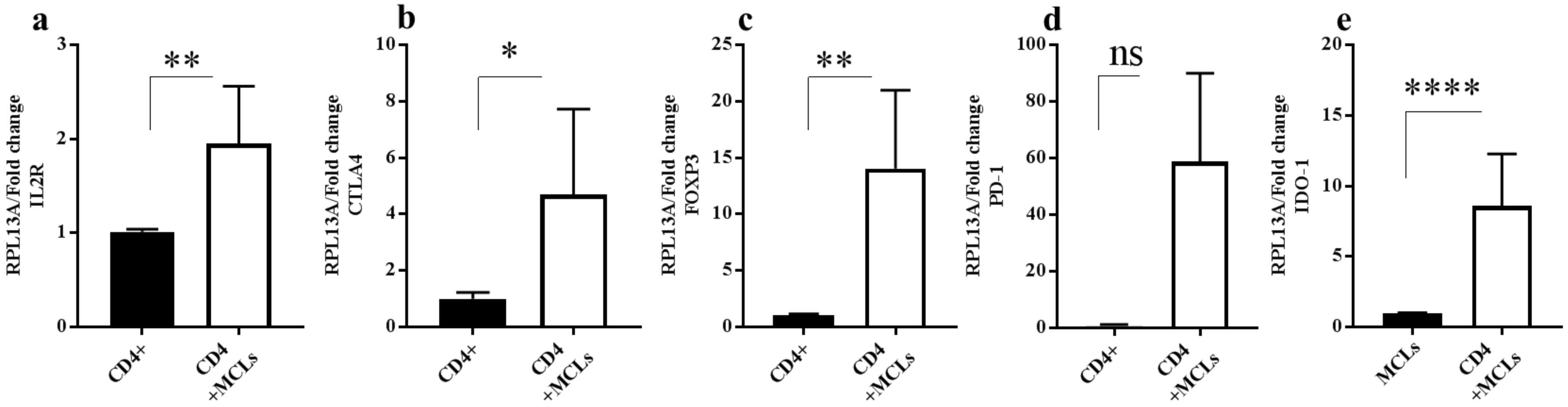

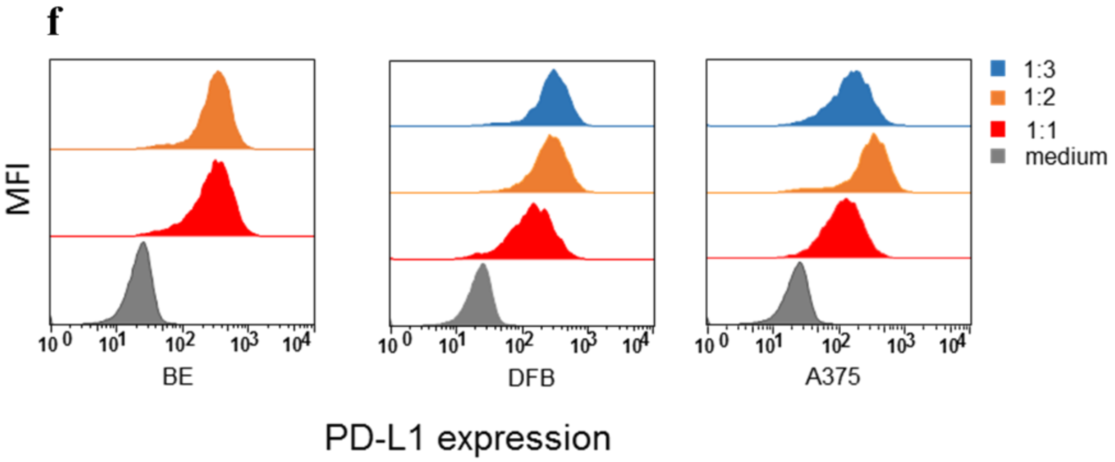

Figure S2. a-e, mRNA expression levels of IL2RA, CTLA4 , FoxP3 and PD1 in re-purified CD4+ T cells and IDO-1 in re-purified MCLs analyzed by real-time PCR and normalized to RPL13A **f,** functional assessment of the blocking IDO1 on MLCs cultured with conditioned medium derived from co-culture experiment of MLCs and CD4+ CD25- T cells treated with INCB024360 (10 nM) in different titration (1:1, 1:2, 1:3) for 5 days. PD-L1 expression were monitored by flow cytometry in MCLs. Graphs show individual data, and horizontal lines show mean ± s.e.m. ****$P \leq 0.0001$, ***$P \leq 0.0005$, **$P \leq 0.001$, *$P \leq 0.05$ by independent samples t-test (two-sided), n = four different melanoma cell lines

Figure S3. Anti-proliferative assessment of the tryptophan depletion by tryptophan titration assay.

a, CFSE-labeled CD4 T cells cultured with four different melanoma cell lines and treated with different doses of the tryptophan (6, 12, 25 and 50 µM) and for 5 days. **b**, a proliferation of CD4+ T-cells in the presence of MLCs alone and with the IDO-1 blockade. CD4+ T cell proliferation observed by flow cytometry. Graphs show individual data, and horizontal lines show mean ± s.e.m.

c-f, KYN, KYNA, 3HK and AA production by MCLs treated with IFNγ (50ng) and TNFα (10ng). **g-h**,IDO1 and KMO expression by MCLs treated with IFNγ (50ng) analyzed by real-time PCR and normalized to RPL13A. Graphs show individual data, and horizontal lines show mean ± s.e.m. *P ≤ 0.05 by independent samples t test (two-sided), n = four different melanoma cell lines.

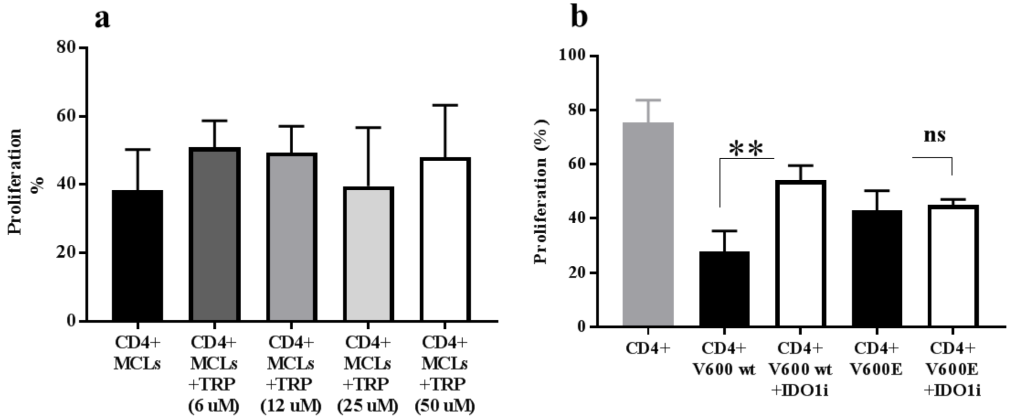

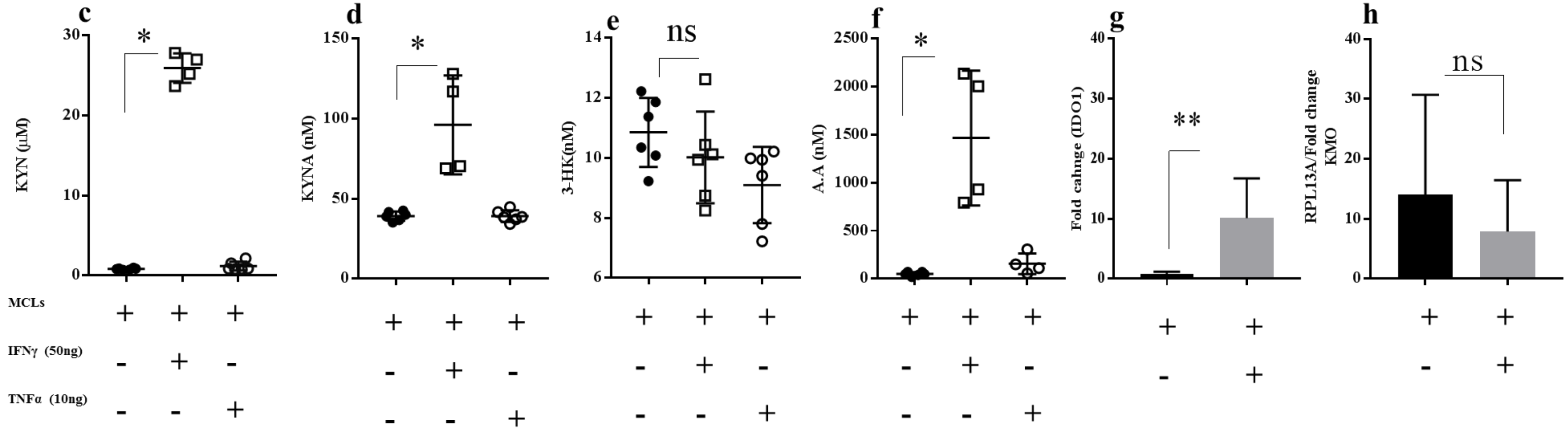

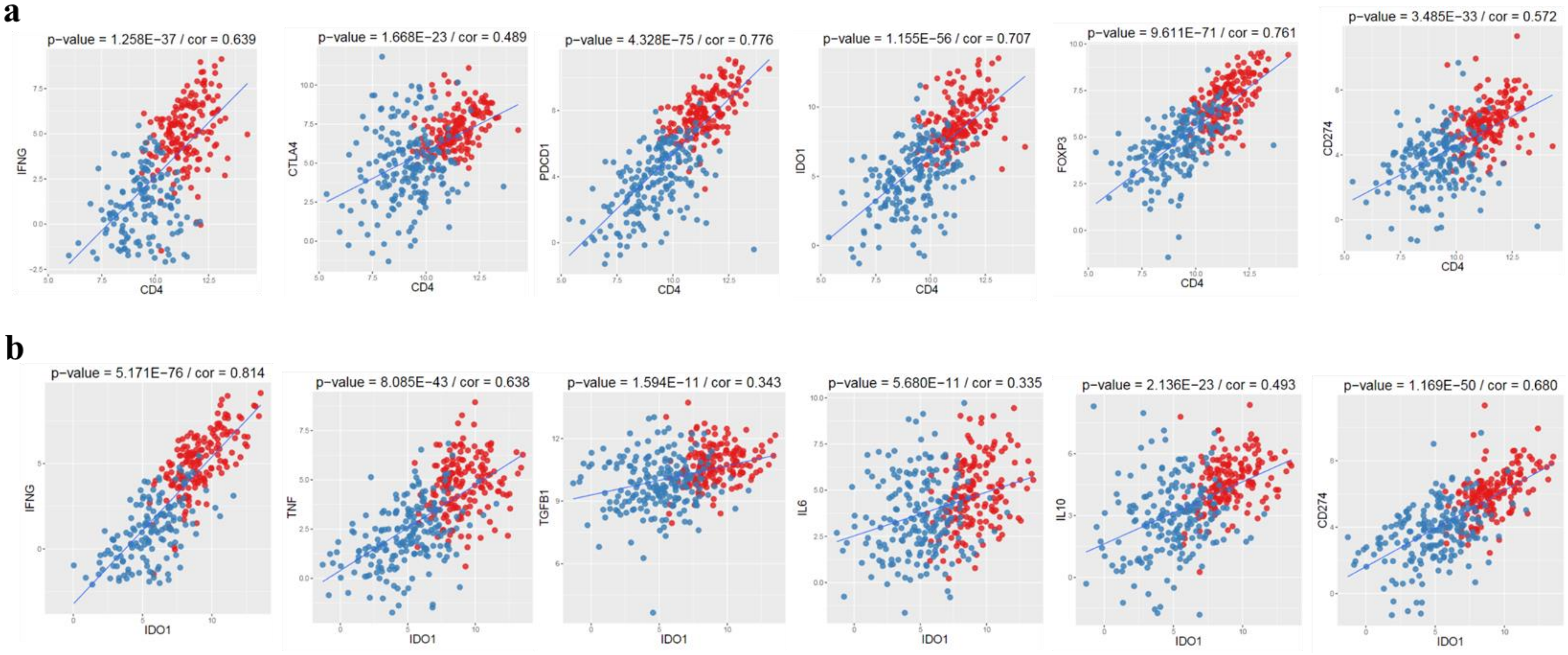

Figure S4. Gene expression data SKCM, TCGA were analyzed through pairwise comparisons .The matrices of scatterplots indicate the correlation of each comparison. **a**, Pearson correlation of CD4+ expression with IFNγ, CTLA4, IDO1, Foxp3 and CD274 (red indicates T-cell-signature high, blue indicates T-cell-signature low). **b**, Pearson correlation of IDO1 expression with IFNγ, TNFα, TGFβ, IL-6, IL-10 and CD274 (PD-L1) (red indicates T-cell-signature high, blue indicates T-cell-signature low).

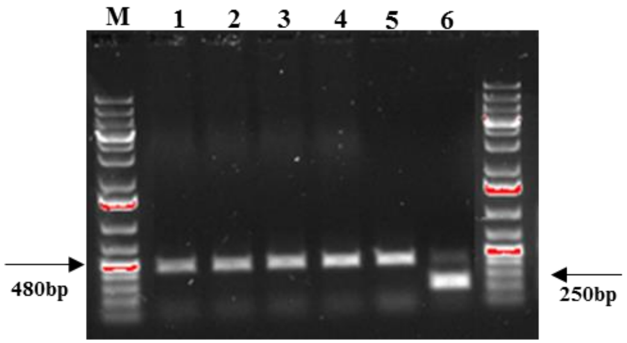

Figure S5. Mycoplasma PCR Detection performed by LookOut Mycoplasma PCR Detection kit, Sigma Aldrich, MP0035. Lane M, amplicon size marker. Bands of 250 bpare characteristic of Mycoplasma, lane 1, negative control; Lanes 2 ,3,4 and5 are BE, DFB, A375 and Skmel-28 cell lines; Lane 6, positive control.

a b

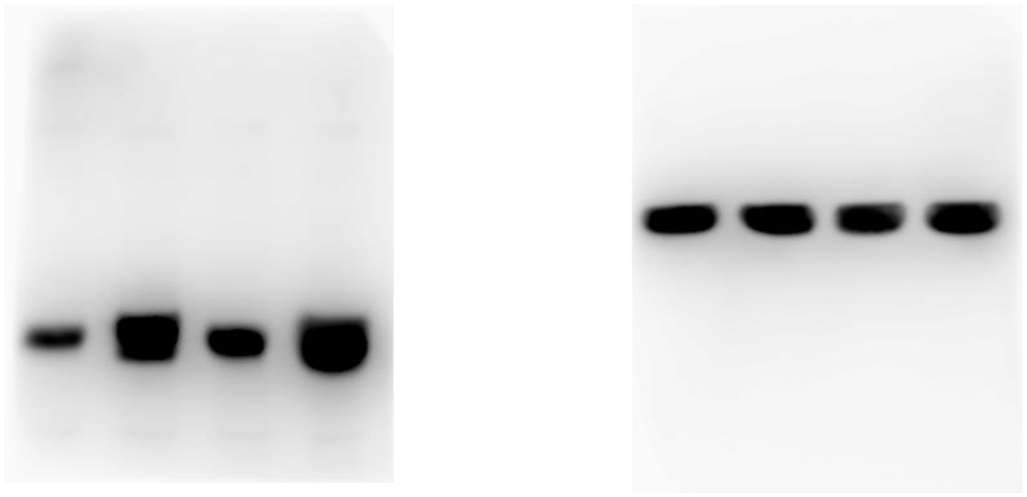

Figure S6. The corresponding image from immunoblotting. a, BRAF over expression (BRAF) **b,** BRAF over expression (actin)
The images are not cropped or adjusted in any manner. The exposure time for the figure is three seconds for BRAF and β-actin.
